# Chronic 5-Aminoimidazole-4-Carboxamide-1-β-d-Ribofuranoside Treatment Induces Phenotypic Changes in Skeletal Muscle, but Does Not Improve Disease Outcomes in the R6/2 Mouse Model of Huntington’s Disease

**DOI:** 10.3389/fneur.2017.00516

**Published:** 2017-09-27

**Authors:** Marie-France Paré, Bernard J. Jasmin

**Affiliations:** ^1^Faculty of Medicine, Department of Cellular and Molecular Medicine, University of Ottawa, Ottawa, ON, Canada

**Keywords:** Huntington’s disease, muscle, exercise mimetics, 5-aminoimidazole-4-carboxamide-1-β-d-ribofuranoside, neurodegeneration

## Abstract

Huntington’s disease (HD) is an autosomal dominant neurodegenerative genetic disorder characterized by motor, cognitive, and psychiatric symptoms. It is well established that regular physical activity supports brain health, benefiting cognitive function, mental health as well as brain structure and plasticity. Exercise mimetics (EMs) are a group of drugs and small molecules that target signaling pathways in skeletal muscle known to be activated by endurance exercise. The EM 5-aminoimidazole-4-carboxamide-1-β-d-ribofuranoside (AICAR) has been shown to induce cognitive benefits in healthy mice. Since AICAR does not readily cross the blood–brain barrier, its beneficial effect on the brain has been ascribed to its impact on skeletal muscle. Our objective, therefore, was to examine the effect of chronic AICAR treatment on the muscular and neurological pathology in a mouse model of HD. To this end, R6/2 mice were treated with AICAR for 8 weeks and underwent regular neurobehavioral testing. Under our conditions, AICAR increased expression of PGC-1α, a powerful phenotypic modifier of muscle, and induced the expected shift toward a more oxidative muscle phenotype in R6/2 mice. However, this treatment failed to induce benefits on HD progression. Indeed, neurobehavioral deficits, striatal, and muscle mutant huntingtin aggregate density, as well as muscle atrophy were not mitigated by the chronic administration of AICAR. Although the muscle adaptations seen in HD mice following AICAR treatment may still provide therapeutically relevant benefits to patients with limited mobility, our findings indicate that under our experimental conditions, AICAR had no effect on several hallmarks of HD.

## Introduction

Huntington’s disease (HD) is an autosomal dominant neurodegenerative genetic disorder characterized by motor, cognitive, and psychiatric symptoms (1). Disease progression typically results in death 15–20 years following onset (2). The HD pathology is the result of expanded CAG trinucleotide repeats in exon 1 of the huntingtin (*HTT*) gene, which results in a polyglutamine [poly(Q)] expansion in the huntingtin protein (HTT) (1, 3). While the function of HTT remains to be fully characterized, it is known to be expressed in multiple tissues (4, 5). As a result of the CAG expansion, mutant HTT proteins form aggregates in a number of tissues (6, 7) and represent a hallmark of the disease. Clinical features include dysphasia, chorea, loss of executive function, personality changes, depression, and anxiety (1). No curative treatments exist for HD, with symptom management currently being the main treatment strategy (8).

It is well established that physical activity supports brain health, greatly benefiting cognitive function, mental health, as well as brain structure and plasticity (9–11). Human trials have shown that both short bouts of endurance exercise and chronic endurance training positively affect performance in cognitive tasks; an effect observed with various types of activity (12–14). In conjunction with cognitive improvements, work on animal models has further revealed that exercise is associated with structural and functional changes in the brain (15). This includes increased brain mass and formation of new, more complex neuronal connections (16–18). Importantly, exercise is associated with adult neurogenesis (18, 19).

Despite some work in this area, current evidence on the effect of exercise in HD remains somewhat equivocal with studies reporting either benefits or detrimental effects. Clinical trials suggest that exercise programs are safe and feasible in HD populations (20, 21). Work conducted with HD animal models has revealed cognitive benefits and neuroprotective effects (22–25). More specifically, voluntary wheel running was found to have motor and cognitive benefits in a mouse model of HD (22), whereas forced treadmill running induced cell proliferation in the dentate gyrus and improved short-term memory (24). Nevertheless, other groups have found either no effect of training programs in animal models of HD (26, 27), or even a worsening of the phenotype (28).

Exercise mimetics (EMs) are a loosely defined group of drugs and small molecules, so called because they target signaling pathways known to be activated by endurance exercise in skeletal muscle (29–31), thereby inducing skeletal muscle adaptations similar to those seen after regular exercise training. Amongst EMs, 5-aminoimidazole-4-carboxamide-1-β-d-ribofurosanide (AICAR) is an activator of AMP kinase (AMPK), and its chronic administration upregulates expression of oxidative genes, increases mitochondrial biogenesis, increases fatty acid β-oxidation, and can cause a shift in muscle fiber types toward slower ones (29, 30). In recent years, AICAR has received considerable attention not only because of its ability to modulate the muscle phenotype but also because of its ability to ameliorate disease progression and outcomes in Duchenne muscular dystrophy and myotonic dystrophy type 1 (32–36).

Converging lines of evidence indicate that EMs may also be beneficial for brain health. Indeed, short-term AICAR treatment enhances spatial memory and hippocampal neurogenesis (37), while also being capable of upregulating pro-survival gene expression in the brain (38). Moreover, AICAR treatment can improve depression-like behaviors as efficiently as treadmill running in a mouse model of depression-like and insulin-resistant state (39). Finally, in α-synuclein overexpressing cells, an *in vitro* model of Parkinson’s disease, AICAR treatment was also shown to offer neuroprotective effects (40).

Little work has been done on the effect of EM treatment in models of HD. Nonetheless, most studies in this area have focused on the use of metformin, a known indirect activator of AMPK (41). For example, metformin administration was shown to be protective in nematode and mouse cell models of HD (42, 43). In the R6/2 mouse model of HD, metformin conferred some benefits on disease progression and survival (44). Unlike metformin, AICAR has very low permeability across the blood–brain barrier (43, 45, 46) and, accordingly, its effects on the brain have been purported to be mediated by peripheral mechanisms (37, 38, 47, 48). In support of this, neurological benefits seen in healthy mice following AICAR treatment were negated in muscle-specific AMPK-deficient animals (47).

Given that AICAR is known to induce neurological benefits (29, 38, 47, 48), as well as positive adaptations in skeletal muscle that mimic those resulting from exercise (29–31), it thus represents a promising therapeutic to examine the impact of AMPK activation in skeletal muscle on HD muscle and brain. The objective of this study was to therefore examine the effect of chronic AICAR treatment on muscle phenotype and disease progression. Specifically, we characterized the effect of AICAR on the muscle phenotype and on disease progression in R6/2 mice.

## Materials and Methods

### Animals and Treatments

B6CBA-Tg(HDexon1)62Gpd/3J transgenic mice (commonly referred to as R6/2 mice, Jackson Laboratories) were used as a model of HD. Although various mouse models of HD have been used in the past, this model recapitulates the severe progressive neurological phenotype observed in humans (49, 50). These animals are transgenic for the 5′ end of the human huntingtin gene and carry approximately 120 ± 5 CAG repeats (49). A male wild-type (WT) mouse was bred to a WT female having received an ovarian transplant from an R6/2 female. The resulting offspring was genotyped using the following primers: 5′-CGCAGGCTAGGGCTGTCAATCATGCT-3′ and 5′-TCATCAGCTTTTCCAGGGTCGCCAT-3′ (51).

Male R6/2 mice and their WT littermates were used in this study and were randomly assigned as follows: WT/SAL *n* = 6, WT/AICAR *n* = 8, R62/SAL *n* = 5, and R62/AICAR *n* = 7. Mortality throughout the study resulted in decreased animal numbers in later timepoints. Specifically, prior to the end of neurobehavioral testing, one animal died in the R62/SAL group, and three died in the R6/2 AICAR group.

Mice were treated with daily subcutaneous AICAR injections at a dose of 500 mg/kg (Toronto Research Chemicals) or a saline control, from 4 to 12 weeks of age. The AICAR dose and treatment duration were selected based on previous optimization in our laboratory, with positive muscle adaptations as primary outcome measures (33, 35). All animals were group-housed and given *ad libitum* access to water and standard lab rodent chow. Mice were determined to have reached humane endpoints at 20% weight loss, or when showing signs of severe distress. Upon reaching 12 weeks of age or humane endpoints (whichever came first), animals were euthanized. All animal procedures were approved by the University of Ottawa Animal Care Committee and were in accordance with the Canadian Council of Animal Care Guidelines.

### Neurobehavioral Tests

Progression of the HD phenotype was quantified using the open field and accelerating rotarod tests (52). In the open field test, mice were placed in a 45 cm × 45 cm × 45 cm box for 10 min while their movement was video recorded and analyzed (Noldus, Ethovision). In the accelerating rotarod test, mice were placed on a rotating textured rod divided into five lanes (IITC Life Sciences). The rod rotation speed increased from 0 to 45 rpm within 1 min, with the maximal test duration set at 2 min. The latency to fall off the rod was recorded.

### Histology and Immunohistochemistry

Following dissection, brains were post-fixed in 5% formalin for 24 h and stored in a 30% glucose and 0.1% sodium azide solution for cryoprotection. Immunostaining for aggregated huntingtin (mEM48, Millipore, MAB5374, 1:100 dilution) was performed on 40 μm-thick brain sections using a DAB peroxidase substrate kit (Vector Laboratories).

Tibialis (TA) anterior muscle samples were dissected and immediately embedded in OCT compound (TissueTek), frozen in isopentane pre-chilled in liquid nitrogen, and stored at −80°C. Ten micrometer-thick muscle cross-sections were used for hematoxylin and eosin (H&E) staining, succinate dehydrogenase (SDH) staining, and immunofluorescence. H&E staining was performed as previously described (32), and the percent of fibers displaying central nucleation was quantified visually on three to four cross-sectional fields of view and expressed as a percentage of the total fibers in those cross-sections. SDH staining was performed as previously described (53), and SDH activity was quantified as the gray intensity on three to four cross-sectional fields of view and expressed as percent absorbance. Additionally, SDH staining was quantified by visually classifying fibers as light, mid-tone, or dark.

Immunofluorescent staining of myosin heavy chain (MHC) isoforms and mutant huntingtin (mHTT) was performed using the M.O.M kit (Vector Laboratories) according to the manufacturer’s specifications. Sections were incubated with undiluted primary antibodies against MHC type I, type IIA, and type IIB (BA-F8, SC-71, and BF-F3, respectively, Developmental Studies Hybridoma Bank) or mHTT (mEM48, Millipore, MAB5374, 1:100 dilution), and a Texas red streptavidin-conjugated secondary antibody (Vector Laboratories) was used for visualization. Co-staining with a primary antibody against laminin (Sigma, L9393, 1:800 dilution) was used to visualize the sarcolemma. Slides were mounted with Vectashield mounting medium with DAPI (Vector Laboratories) in order to visualize the nuclei.

All slides were imaged with a Zeiss AxioImager M2 microscope equipped with the Zeiss AxioCam MRm detector. Image analysis was conducted using ImageJ (National Institutes of Health).

### RNA Extraction and Reverse Transcription (RT)-Quantitiative PCR (qPCR)

Snap-frozen muscles were pulverized on dry ice. RNA was extracted from the muscle powder using TRIzol reagent (Invitrogen) as previously described (32), and treated with DNAse to mitigate DNA contamination (Ambion). RT was performed in a solution containing 5 mM MgCl_2_, 1× PCR buffer, 1 mM dNTP, 1 U/mL RNase inhibitor, 5 U/mL Moloney murine leukemia virus reverse transcriptase (Applied Biosystems), and 2.5 mM random hexamers (Invitrogen). The MX3005p real-time PCR system (Stratagene) and a QuantiTect SYBR Green PCR kit (Qiagen) were used to carry out real-time qPCRs. The primers used are as follows: *Murf1* forward 5′-TGTCTGGAGGTCGTTTCCG-3′, *Murf1* reverse 5′-ATGCCGGT CCATGATCACTT-3′, *Mafbx* forward 5′-AGCGACCTCAG CAG TT ACTGC-3′, Mafbx reverse 5′-CTTCTGGAATCCAGGATGGC-3′.

### Immunoblotting

Pulverized muscle samples were suspended in 300 µL RIPA buffer (150 mM NaCl, 1% NP-40, 0.1% SDS, 50 mM Tris–HCl, and 12 mM sodium deoxycholate, pH 7.4) containing protease and phosphatase inhibitors (Complete Cocktail and PhosphoStop, Roche) (54). Protein concentration was determined using a bicinchoninic acid protein assay (Thermo Fisher). 10–30 µg of total protein was separated through SDS-PAGE and transferred onto PVDF membranes (Immobilon, Millipore). Membranes were blocked with 5% skim milk in TBS-Tween, followed by incubation in primary antibodies for 1 h at room temperature or overnight at 4°C. The following primary antibodies were used in these experiments: AMPK (Cell Signaling, #2532, 1:500), p-AMPK (Cell Signaling, #2535, 1:500), PGC-1α (Abcam, ab54481, 1:500), MuRF1 (ECM Biosciences, MP3401, 1:1,000), MAFbx (ECM Biosciences, AP2041, 1:1,000), and oxidative phosphorylation (OXPHOS) cocktail (Mitosciences, ab110413, 1:1,000). Following washing with TBS-Tween, membranes were incubated in the appropriate HRP-conjugated secondary antibodies (Jackson ImmunoResearch) for 1 h at room temperature and visualized using ECL reagents (Thermo Scientific) and autoradiography on X-ray film (Bio-Rad). Lastly, membranes were stained with ponceau red to confirm equal loading. Quantification was performed using ImageLab software (Bio-Rad).

### Statistical Analyses

All data were analyzed using Student’s *t*-tests, two-, and three-way ANOVAs, as appropriate, and the Tukey’s LSD as a *post hoc* test. Survival data were analyzed using Kaplan–Meier survival analysis. GraphPad Prism software was used to perform these analyses, with the exception of the three-way ANOVAs, which were performed using SPSS. Data are presented as mean ± SEM. A *P*-value of <0.05 was considered statistically significant.

## Results

### AICAR Upregulates PGC-1α Expression and Alters the Muscle Phenotype

**Figure 1:**
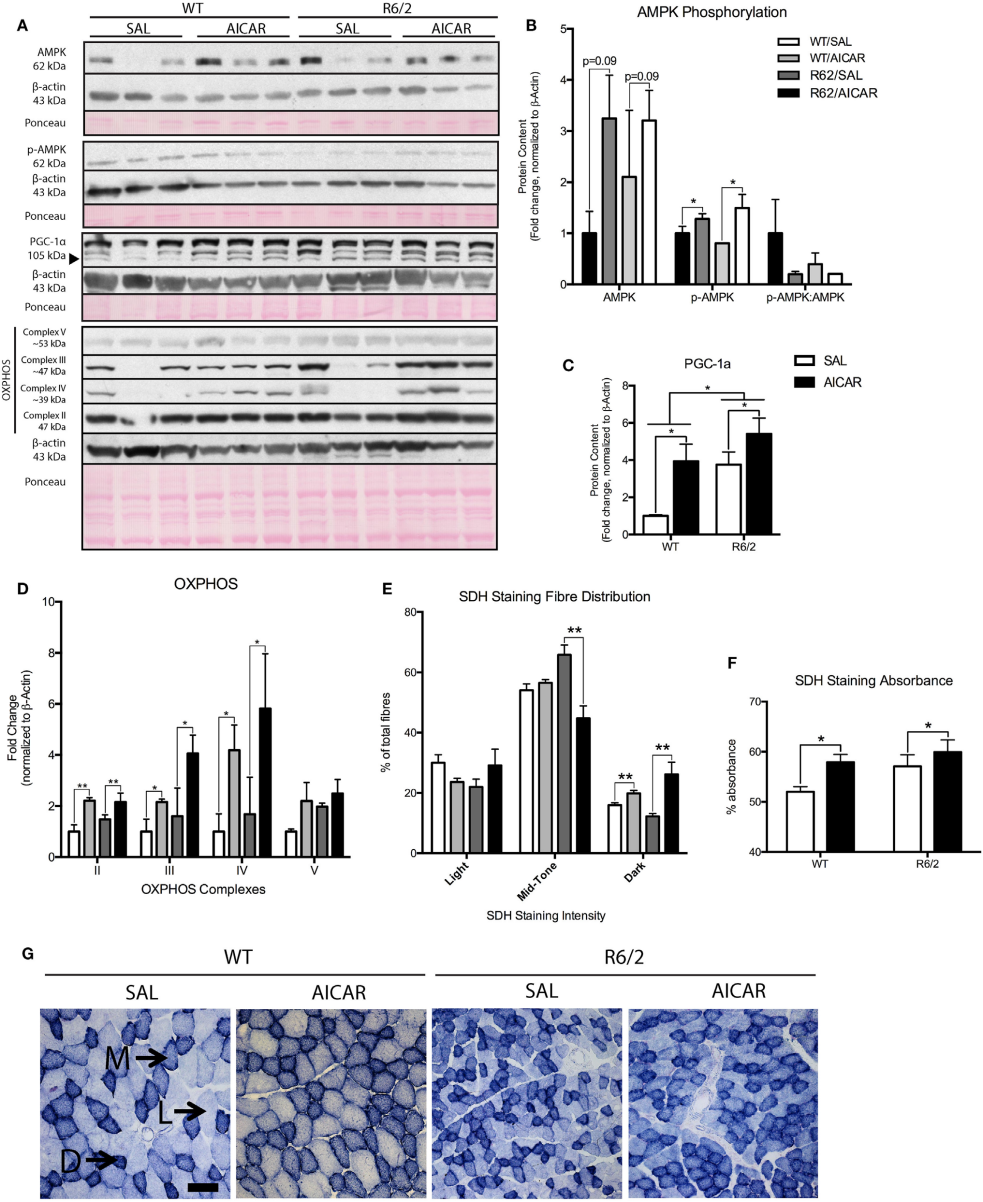
5-Aminoimidazole-4-carboxamide-1-β-d-ribofuranoside (AICAR) upregulates PGC-1α expression and alters the muscle phenotype. **(A)** Representative immunoblots of AMP kinase (AMPK), p-AMPK, PGC-1α, and complexes of the oxidative phosphorylation (OXPHOS) chain. β-actin and ponceau staining are shown to confirm equal loading and effective transfer. **(B)** Quantification of AMPK and p-AMPK immunoblots in the gastrocnemius (GAST) muscle of SAL- and AICAR-treated wild-type (WT) and R6/2 mice. The calculated p-AMPK:AMPK ratio is also shown. While AICAR increased both AMPK and p-AMPK protein content, the p-AMPK:AMPK ratio was unchanged. **(C)** Quantification of a PGC-1α immunoblot in the GAST muscle of SAL- and AICAR-treated WT and R6/2 mice. PGC-1α expression was increased in the R6/2 and in AICAR-treated animals. **(D)** Quantification of OXPHOS immunoblots in the GAST muscle of SAL- and AICAR-treated WT and R6/2 mice. AICAR increased the expression of complexes II, III, and IV. **(E)** Quantification of light, mid-tone, and dark succinate dehydrogenase (SDH)-stained fibers, expressed as a percentage of total fibers. AICAR decreased the number of mid-tone stained fibers in the R6/2 and increased the number of dark-stained fibers in both genotypes. **(F)** Quantification of SDH staining intensity, expressed as percent absorbance. AICAR-treated muscle displayed greater (*P* < 0.05) overall staining intensity than saline-treated controls. **(G)** Representative images of SDH staining in SAL- and AICAR-treated WT and R6/2 mouse tibialis muscle cross-sections. Scale bar indicate 50 µm, annotations indicate light (L), mid-tone (M), and dark **(D)** fibers. Data shown as mean ± SEM, *n* = 3–8; *P < 0.05, **P < 0.01.

Given that AICAR is a known activator of the AMPK pathway (30), we first assessed activation of AMPK in skeletal muscle through phosphorylation at the Thr172 site. Total AMPK [*F*(1,8) = 3.798, *P* = 0.09] and p-AMPK [*F*(1,8) = 9.223, *P* < 0.05] protein levels were increased in AICAR-treated WT and R6/2 mice (Figures 1A,B). However, the increased expression of AMPK in AICAR-treated WT and R6/2 mice negated a potential increase in the ratio of p-AMPK to AMPK in these groups (Figures 1A,B). This is consistent with previous reports showing modest effects of AICAR on AMPK phosphorylation in muscle following chronic administration (55, 56), a phenomenon likely caused by habituation. Of relevance, we did not observe an effect of genotype in either AMPK or p-AMPK protein content in muscle from WT and R6/2 animals following AICAR administration, indicating that HD mice responded to treatment similarly to WT animals.

We next measured protein levels of PGC-1α, a known downstream target of AMPK signaling (57). Interestingly, expression of PGC-1α was significantly higher [*F*(1,8) = 8.804, *P* < 0.05] in muscles from R6/2 mice compared with controls (Figures 1A,C). Moreover, PGC-1α levels were significantly [*F*(1,8) = 10.43, *P* < 0.05] increased in both WT and R6/2 mouse muscles following AICAR (Figures 1A,C). Such increases in the protein content of PGC-1α in skeletal muscle demonstrate activation of the AMPK signaling pathway with AICAR treatment (57).

As AICAR induces important changes in oxidative metabolism in muscle (29, 30), we also determined the expression pattern of various OXPHOS complexes. While no effect of genotype was observed, we found that AICAR treatment caused a significant increase in complexes II [*F*(1,8) = 15.17, *P* < 0.01], III [*F*(1,8) = 6.628, *P* < 0.05], and IV [*F*(1,8) = 6.603, *P* < 0.05] in muscles from AICAR-treated animals compared with saline-treated controls (Figures 1A,D). To complement these observations, the activity of the enzyme SDH was also monitored in muscles from saline- and AICAR-treated WT and R6/2 mice. Visual quantification of the number of light, mid-tone, and dark SDH-stained fibers revealed that R6/2 mice had a similar staining pattern in their muscles as WT animals (Figures 1E,G). AICAR treatment significantly decreased [*F*(1,8) = 16.55, *P* < 0.05] the number of mid-tone stained fibers in muscles from R6/2 mice compared with saline-treated R6/2 mice. Additionally, AICAR treatment significantly increased [*F*(1,8) = 16.99, *P* < 0.05] the number of dark-stained fibers in muscle from both mouse genotypes (Figures 1E,G). When measured as overall percent absorbance, muscles fibers from AICAR-treated muscle displayed greater [*F*(1,8) = 5.316, *P* < 0.05] overall staining intensity than saline-treated controls (Figures 1F,G). Taken together, these data indicate that chronic AICAR treatment induces a shift toward a more oxidative metabolism in muscle, a well-described effect of this compound (58). Moreover, it appears that muscles from both WT and R6/2 mice responded similarly to the AICAR treatment.

**Figure 2:**
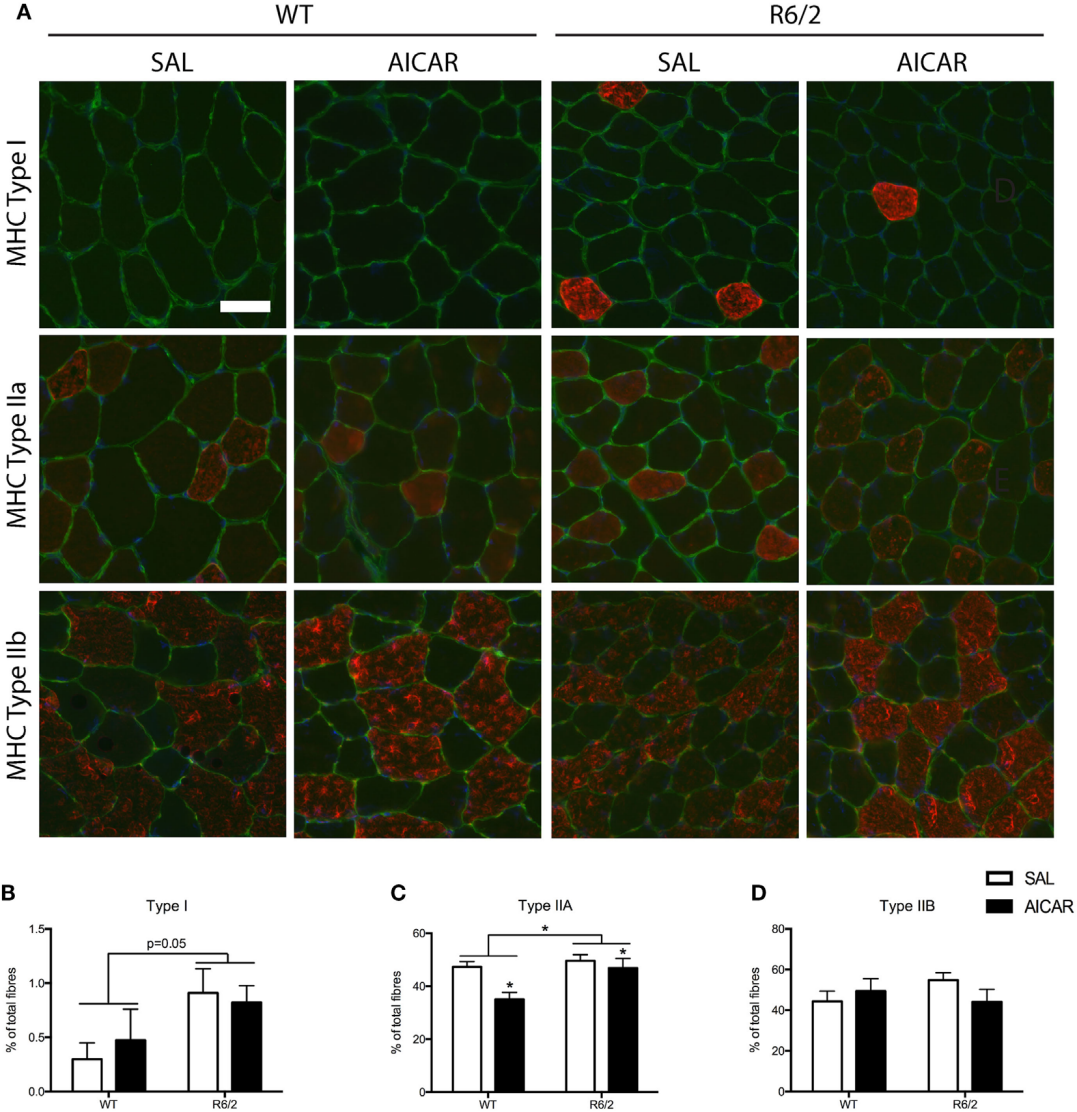
5-Aminoimidazole-4-carboxamide-1-β-d-ribofuranoside (AICAR) modulates the muscle phenotype. **(A)** Representative images of myosin heavy chain (MHC) immunofluorescence in SAL- and AICAR-treated wild-type and R6/2 mouse tibialis muscle cross-sections. Scale bar indicate 50 µm, laminin (green), and DAPI (blue) were used to delineate fiber structure. **(B)** Quantification of the expression of type I MHC, expressed as a percentage of total fibers. R6/2 muscle had a higher percentage of fibers expressing type I MHC. **(C)** Quantification of the expression of type IIA MHC, expressed as a percentage of total fibers. AICAR treatment decreased the percent of fibers expressing type IIA MHC, but R6/2 muscle had a greater number of type IIA positive fibers. **(D)** Quantification of the expression of type IIB MHC, expressed as a percentage of total fibers. Data shown as mean ± SEM, *n* = 3–8; *P < 0.05.

In order to further examine the effect of AICAR on the muscle phenotype, we also examined the fiber-type distribution through MHC immunofluorescence. Treatment with AICAR decreased the percentage of muscle fibers expressing type IIA MHC [*F*(1,8) = 7.507, *P* < 0.05, Figures 2A,C]. The expression of type I and type IIB MHC was unchanged with AICAR treatment, but R6/2 mice had higher expression of type I [*F*(1,8) = 5.127, *P* = 0.05] and type IIA [*F*(1,8) = 6.795, *P* < 0.05, Figures 2A,B,D]. The percentage of fibers expressing type IIB was not significantly different between WT and R6/2 animals. While AICAR is known to increase the activity of oxidative enzymes (as described above), it has been suggested that this phenomenon may not necessarily correlate with corresponding changes in MHC expression (59).

### R6/2 Mice Display Muscle Atrophy

**Figure 3:**
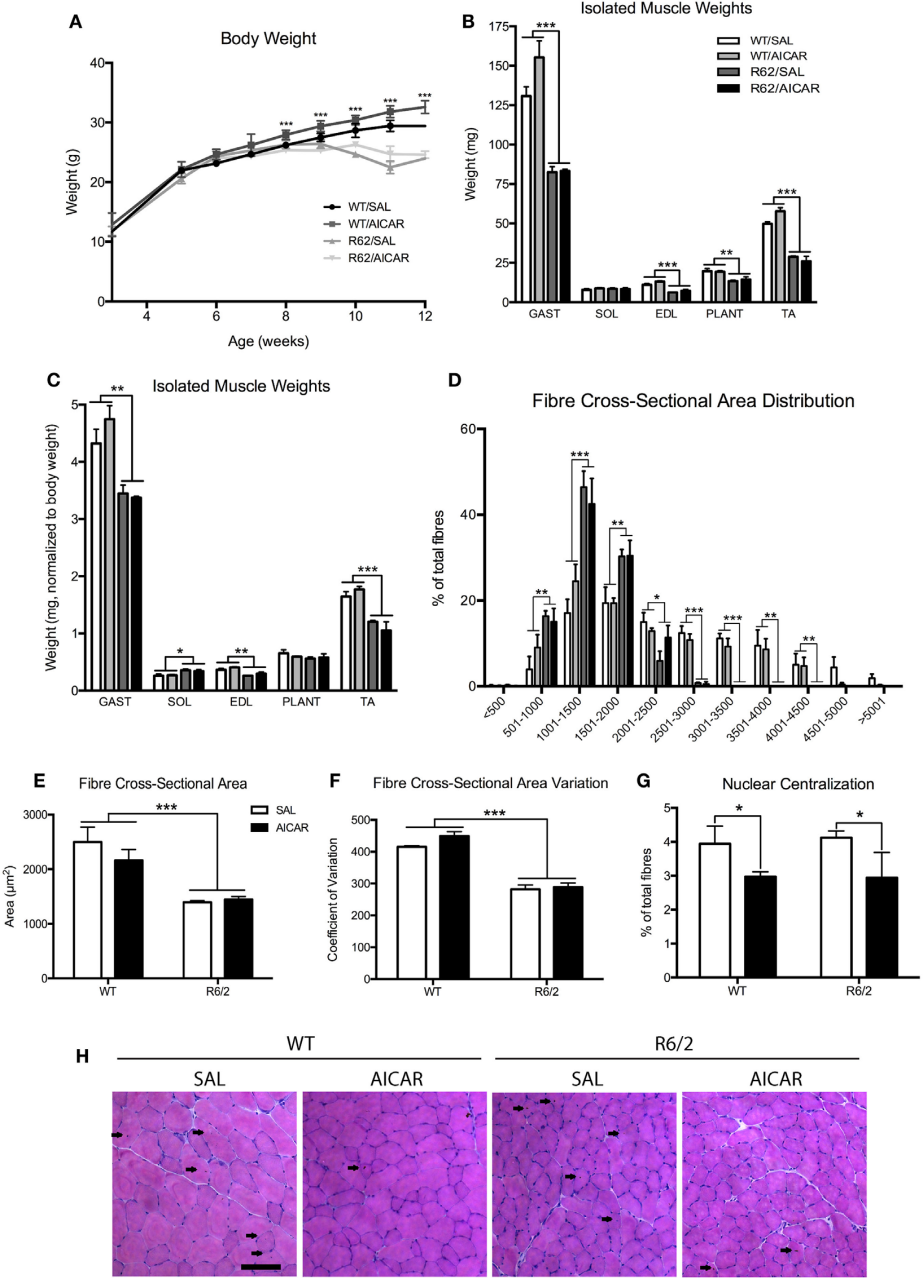
R6/2 mice show muscle atrophy. **(A)** Body weight in SAL- or 5-aminoimidazole-4-carboxamide-1-β-d-ribofuranoside (AICAR)-treated wild-type (WT) and R6/2 mice. Note that significant effects of age are omitted in the interest of clarity. R6/2 animals were significantly smaller than WT controls beyond 8 weeks of age. **(B)** Isolated muscle weights. GAST, gastrocnemius; SOL, soleus; EDL, extensor digitorum longus; PLANT, plantaris; TA, tibialis. GAST, EDL, PLANT, and TA were significantly smaller in R6/2 animals. **(C)** Isolated muscle weights, normalized to body weight. GAST, EDL, and TA were significantly smaller in R6/2 animals. **(D)** Distribution of fiber cross-sectional area, expressed as a percentage of total fibers included in 500 µm brackets. R6/2 animals had significantly fewer large fibers. **(E)** Quantification of total fiber cross-sectional area. R6/2 animals had significant fiber atrophy. **(F)** Coefficient of variation of the cross-sectional area. R6/2 animals had significantly less variation in fiber size. **(G)** Quantification of centralized nuclei, expressed as a percentage of total fibers. AICAR decreased the percentage of fibers with central nucleation. **(H)** Representative images of hematoxylin and eosin staining in TA muscle cross-sections. Scale bar indicates 100 µm, centralized nuclei indicated with arrows. Data shown as mean ± SEM, *n* = 3–8; *P < 0.05, **P < 0.01, ***P < 0.001.

We determined the size of muscle fibers following AICAR treatment of WT and R6/2 mice. First, R6/2 mice were significantly [*F*(1,8) = 71.07, *P* < 0.001] smaller in weight than WT animals from the age of 8 weeks onward (Figure 3A). This progression was not affected by AICAR treatment. Second, all individually weighed muscles of the hindlimb were significantly [*F*(1,12) > 14.12, *P* < 0.001] smaller in R6/2 mice compared with controls (Figure 3B), except for the soleus (SOL) muscle. When normalized to body weight, gastrocnemius (GAST) [*F*(1,12) = 12.17, *P* < 0.01], extensor digitorum longus [*F*(1,12) = 16.76, *P* < 0.01], and TA [*F*(1,12) = 38.54, *P* < 0.001] muscles were still significantly smaller in R6/2 mice compared with WT animals (Figure 3C). None of the muscles weighed were significantly different between saline- and AICAR-treated animals.

Morphologically, muscles from R6/2 mice displayed a more homogenous fiber size distribution, with a dramatic reduction [*F*(1,8) > 16.53, *P* < 0.01] in large fibers. As a result, the overall average muscle fiber cross-sectional area was significantly smaller [*F*(1,8) = 27.72, *P* < 0.001] in R6/2 muscle compared with WT animals (Figures 3D,E). This was further observed as a decrease [*F*(1,8) = 156.4, *P* < 0.001] in the cross-sectional area coefficient of variation (Figure 3F). Neither the fiber cross-sectional area nor the fiber size distribution were affected by AICAR treatment. However, we noted that AICAR decreased [*F*(1,8) = 5.268, *P* < 0.05] the percentage of fibers with centralized nuclei in both WT and R6/2 mice (Figures 3G,H). Collectively, these findings indicate that muscles from R6/2 mice display severe muscle atrophy, an observation consistent with previous results (60–62). Our findings further show that this severe atrophy affects primarily large diameter fibers.

**Figure 4:**
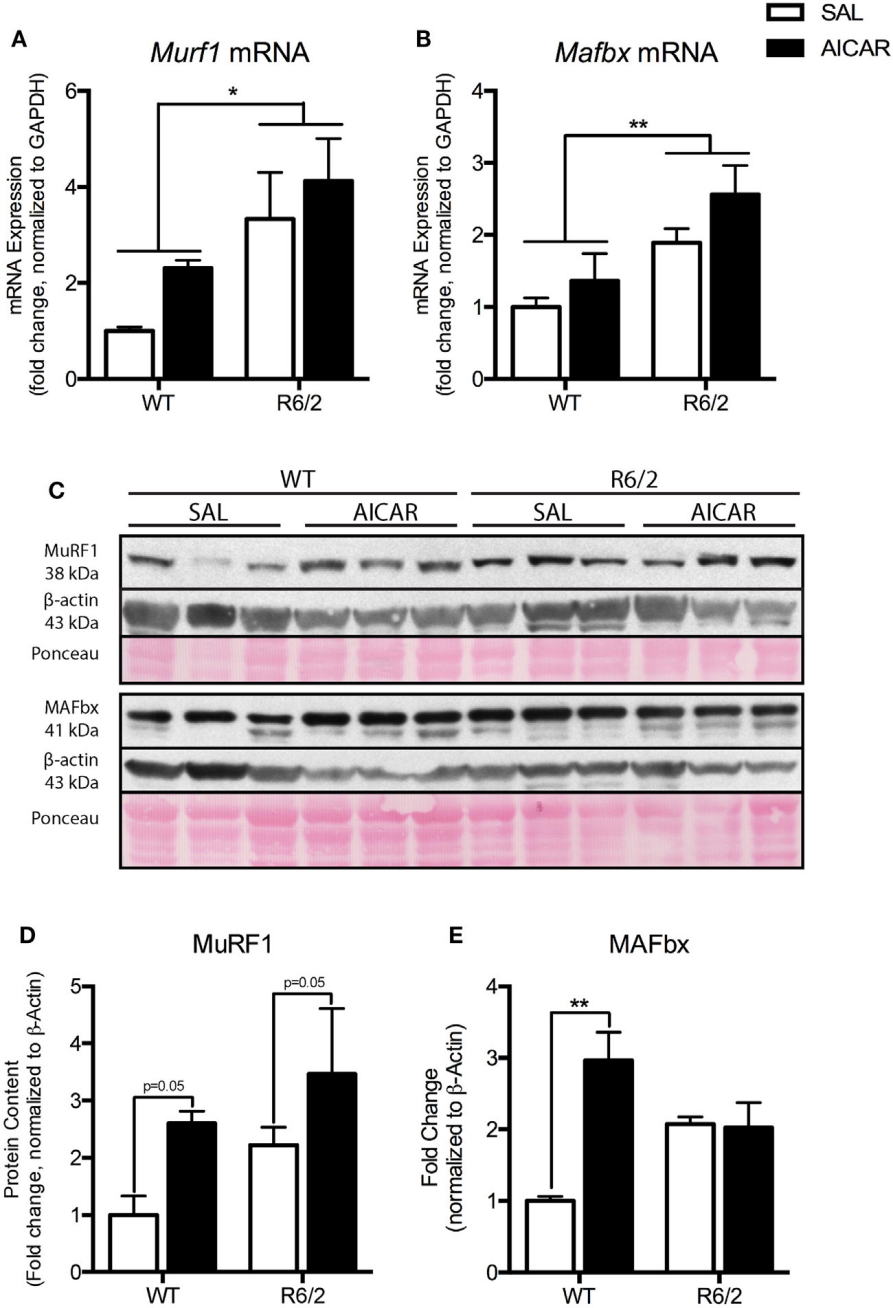
Markers of atrophy are upregulated in R6/2 mouse muscle. **(A)** Expression of *Murf1* mRNA in the gastrocnemius (GAST) muscle of saline (SAL)- and 5-aminoimidazole-4-carboxamide-1-β-d-ribofuranoside (AICAR)-treated wild-type (WT) and R6/2 mice. R6/2 animals had a greater expression of *Murf1* mRNA. **(B)** Expression of *Mafbx* mRNA in the muscle of SAL- and AICAR-treated WT and R6/2 mice. R6/2 animals had a greater expression of *Mafbx* mRNA. **(C)** Representative immunoblots of MuRF1 and MAFbx. β-actin and ponceau staining are shown to confirm equal loading and effective transfer. **(D)** Quantification of an MuRF1 immunoblot in the GAST muscle of SAL- and AICAR-treated WT and R6/2 mice. AICAR increased the protein content of MuRF1. **(E)** Quantification of an MAFbx immunoblot in the GAST muscle of SAL- and AICAR-treated WT and R6/2 mice. AICAR increased the protein content of MAFbx in the WT animals. Data shown as mean ± SEM, *n* = 3; *P < 0.05, **P < 0.01.

Because we observed muscle fiber atrophy in R6/2 animals, we examined the mRNA and protein levels of known atrogenes (63). Both *Murf1* [*F*(1,8) = 9.763, *P* < 0.05] and *Mafbx* [*F*(1,8) = 12.15, *P* < 0.01] mRNA expression were increased in R6/2 mouse muscle compared with WT animals (Figures 4A,B). Similar increases in muscles between WT and R6/2 mice were also seen at the protein level, at least for MuRF1 [*F*(1,8) = 7.492, *P* = 0.05, Figures 4C,D]. In general, AICAR treatment caused an induction of atrogene expression at both the mRNA and protein levels in muscles from all mice (Figures 4A–E). These data are in agreement with previous *in vitro* work showing atrogene induction following AICAR treatment (64–66). Moreover, it appears that the increased expression of atrogenes following AICAR treatment did not exacerbate the atrophy seen in muscle from R6/2 mice.

### AICAR Does Not Mitigate Disease Progression or Improve Survival in R6/2 Mice

**Figure 5:**
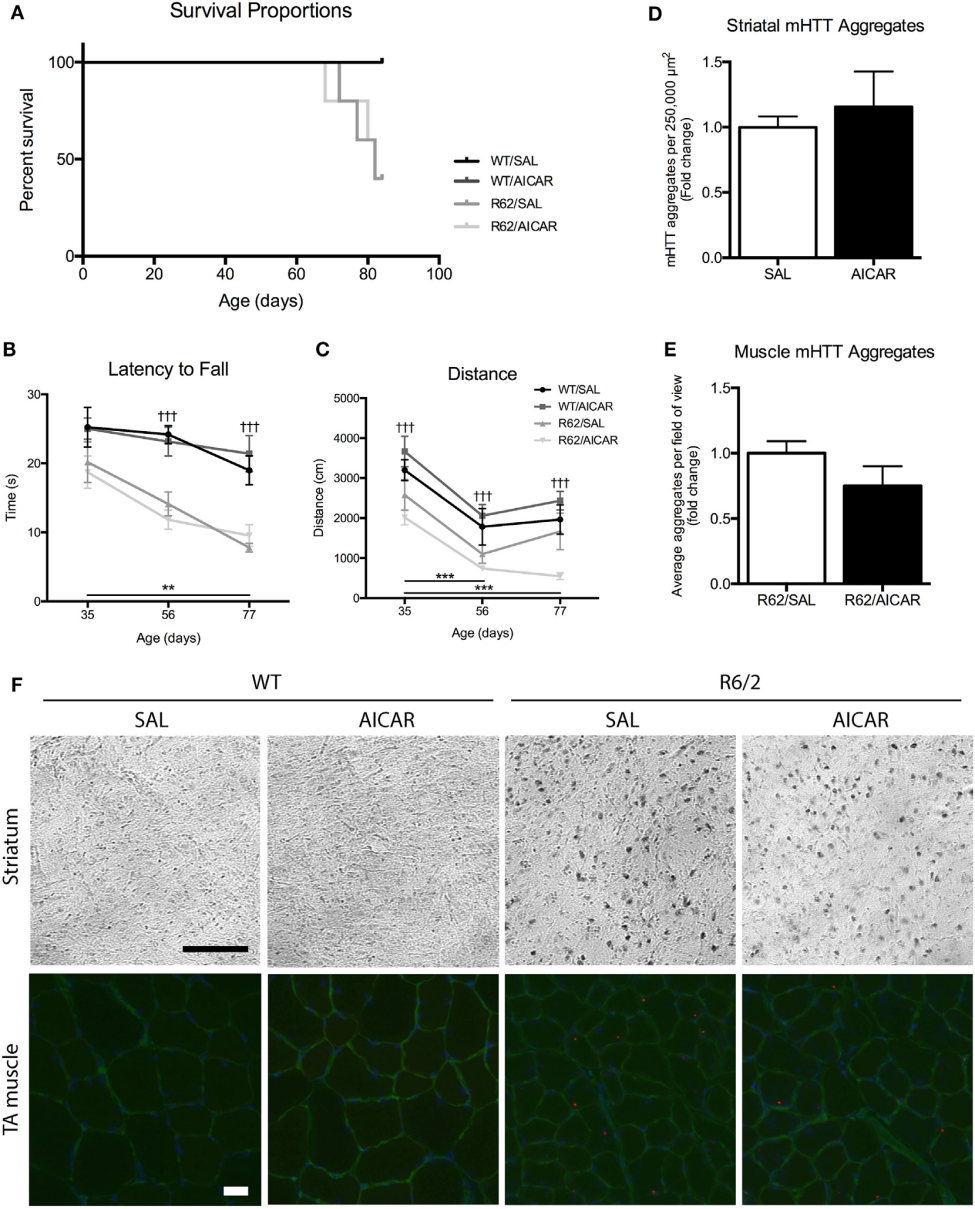
5-Aminoimidazole-4-carboxamide-1-β-d-ribofuranoside (AICAR) does not improve the disease phenotype in R6/2 mice. **(A)** Survival curve for SAL- or AICAR-treated WT and R6/2 mice. Both SAL- and AICAR-treated R6/2 mice had a survival rate of 50%. **(B)** Latency to fall in the accelerating rotarod test. R6/2 animals had a significantly shorter latency to fall at 56 and 77 days, compared with WT. **(C)** Distance traveled in the open field test. R6/2 animals traveled a significantly shorter distance compared with WT at all time points. **(D)** Quantification of the number of mutant huntingtin (mHTT) aggregates per 500 µm × 500 µm area of striatum. There was no difference in number of mHTT aggregates with AICAR treatment. **(E)** Quantification of the average number of mHTT aggregates per field of view in tibialis (TA) muscle cross-sections. There was no difference in number of mHTT aggregates with AICAR treatment. **(F)** Representative images of mHTT (EM48) histochemistry in the striatum and immunofluorescence in TA muscle of SAL- and AICAR-treated wild-type (WT) and R6/2 mice. Histochemistry scale bar indicates 100 µm. Immunofluorescence scale bar indicates 50 µm, laminin (green), and DAPI (blue) were used to delineate fiber structure. Data shown as mean ± SEM, *n* = 3–8; ^†††^*P* < 0.001 vs. WT; **P < 0.01, ***P < 0.001 (effects of age).

A second main objective of this study was to examine the impact of AICAR on HD progression. As shown in Figure 5A, AICAR treatment did not improve the survival of R6/2 mice. In both R6/2 treatment groups, exactly 50% of animals died before the end of the protocol (Figure 5A). Specifically, the R62/SAL and R62/AICAR groups had a mean age at death/euthanasia of 81.4 ± 1.71 and 78.0 ± 4.32 days, respectively. To determine the effect of AICAR on neurobehavioral outcomes, WT and R6/2 mice were subjected to the accelerating rotarod and open field tests at 5, 8, and 11 weeks of age. As expected, R6/2 mice had a shorter latency to fall [*F*(1,11) = 48.31, *P* < 0.001] in the accelerating rotarod test at 8 and 11 weeks of age (Figure 5B), and both genotypes displayed a significant decline [*F*(2,11) = 10.49, *P* < 0.01] in latency to fall between 5 and 11 weeks of age. In agreement with these observations, R6/2 mice traveled a smaller distance [*F*(1,11) = 32.81, *P* < 0.001] in the open field than their WT counterparts at all ages tested (Figure 5C). Both genotypes traveled a smaller [*F*(2,11) = 24.99, *P* < 0.001] distance at 8 and 11 weeks of age compared with the 5-week-old time point. In these experiments, AICAR treatment had no effect on the performance as determined by the rotarod and open field tests. As mentioned in Section “Materials and Methods,” the R6/2 mouse model is prone to sudden mortality, and animal group numbers decreased throughout the neurobehavioral testing period. Overall, R6/2 mice showed the expected neurobehavioral deficits associated with HD, and these were not mitigated by the chronic AICAR treatment.

Finally, immunohistochemistry was performed on brain sections to visualize mHTT aggregation, a hallmark of HD (5). Aggregates were only observed in R6/2 striatal sections, and their abundance was not affected by AICAR treatment (Figures 5D,F). We also performed immunofluorescence to examine mHTT aggregation in skeletal muscle. In TA muscle cross-sections, mHTT immunofluorescence revealed the presence of mHTT puncta only in samples from R6/2 mice. Similar to what we observed in striatal sections, the abundance of these puncta was not affected by AICAR treatment (Figures 5E,F). These analyses were performed on an *n* = 3 for each group.

## Discussion

The purpose of this study was to examine the effects of the EM AICAR on the skeletal muscle and disease phenotype of a mouse model of HD. Under our conditions, AICAR increased expression of PGC-1α and induced the expected shift toward a more oxidative skeletal muscle phenotype. However, the treatment failed to induce benefits on HD progression. Indeed, neurobehavioral deficits, mHTT aggregate density, as well as muscle atrophy were not mitigated by the chronic administration of AICAR. Although the skeletal muscle adaptations seen in HD mice may still provide therapeutically relevant effects for patients with limited mobility, our findings suggest that AICAR has essentially no effect on several hallmarks of HD, at least under the conditions of our study.

### AICAR Treatment Induces a More Oxidative Phenotype in HD Muscle

In the current study, AICAR treatment had the expected effects on muscle phenotype (29, 30), upregulating total and phosphorylated AMPK content as well as the downstream effector PGC-1α. AMPK activation through phosphorylation occurs rapidly in response to energy imbalance, but this has longer-term effects through downstream regulation of transcription (67). This may explain the notable increase in PGC-1α content in the absence of an increase in the ratio of phosphorylated to total AMPK. Overall, AICAR treatment did not cause a fiber-type shift but resulted in a marked increase in SDH activity indicative of a more oxidative phenotype. This is in agreement with the known fact that AICAR increases oxidative enzyme activity (58), independently of a shift in the expression of MHC isoforms (59). In support of this greater oxidative metabolism, the expression of several OXPHOS complexes was found to be increased following AICAR treatment.

While this study is the first to examine the effects of AICAR in HD muscle, Chaturvedi and colleagues investigated AMPK function in a mild HD mouse model by depleting ATP using β-guanidinopropionic acid (GPA). In this study, it was observed that total AMPK levels were reduced in HD muscle, and were unresponsive to GPA treatment (68). Contrary to these findings, we found that AICAR treatment had the same effect in muscles from R6/2 mice as in those from WT littermates, suggesting that pharmacological activation of AMPK in muscle might still provide benefits to HD patients having limited exercise capacity.

### AICAR Does Not Mitigate Atrophy in HD Muscle

Huntington’s disease is known to be characterized by profound muscle atrophy (60–62, 69), and the present study corroborates these findings of overall muscle mass loss. Of note, this general atrophic pattern seems to spare the slow SOL muscle, which is a postural muscle. The preferential atrophy of fast muscles in this case is likely due to a reduction in locomotion in R6/2 mice, but with more constant recruitment of the SOL necessary to maintain posture (70). In our work, we further report for the first time that in parallel to this loss of muscle mass, fiber cross-sectional area distribution in HD mice is dramatically affected leading to a more homogeneous fiber size compared with WT animals. AICAR is known to increase running endurance (30) and to protect from muscle atrophy in a number of muscle disorders (56, 71, 72). For example, AICAR mitigates muscle atrophy associated with spinal muscular atrophy, muscular dystrophy, and cancer cachexia (56, 71, 72). However, our group and others have found that the protection from atrophy is not universal (33, 73). Indeed, in the current study, we found no beneficial effect of AICAR treatment on body weight, muscle mass, and fiber cross-sectional area.

The ubiquitin-proteasome system (UPS) is an important contributor to muscle degradation under atrophic conditions, and its components (referred to as atrogenes) are regulated by the FoxO family of transcription factors (74). E3 ubiquitin ligases, such as MuRF1, are the final step in tagging target proteins with ubiquitin for degradation (63, 75). In HD R6/2 mice, MuRF1 protein content was shown to be upregulated (60), which concords with our measures of *Murf1* mRNA. At the protein level, MuRF1 was elevated in HD muscle, and these levels were further upregulated following AICAR treatment. AICAR is known to activate FoxO downstream of AMPK, which results in transcriptional upregulation of atrogenes. This has been shown both *in vitro* and *in vivo* (64, 66, 76), and such a pattern of regulation is confirmed by our measures of MuRF1. In contrast, previous work has shown that PGC-1α is protective against atrophy through suppression of FoxO signaling (77). For example, AICAR treatment in a model of cancer cachexia was found to be beneficial through a downregulation of atrogene expression (72). Interestingly, our measures of fiber cross-sectional area indicate that while AICAR upregulated expression of atrogenes, it did not further exacerbate the atrophy seen in HD muscle. This paradox may be explained by the role of MuRF1 and MAFbx in protective muscle remodeling following disease or disease-related atrophy (74), and/or by the increased expression of PGC-1α that we noted following AICAR administration. In addition, there is evidence that the relationship between AMPK signaling and the UPS is not unidirectional. It has been suggested that AMPK is regulated by the UPS through ubiquitin modifications (78), and that ubiquitinated proteins may activate AMPK by altering the energy status of the cell (79). This may point to a compensatory mechanism in the UPS in response to the AICAR-associated increase in AMPK content.

### AICAR Does Not Improve the Progression of HD

AMP kinase plays a central role in the regulation of energy homeostasis by targeting several downstream effectors (80). Because of its role in promoting longevity and combating cellular stress (81), AMPK has been of interest in the context of HD. Previous work by Ju et al. suggested that striatal AMPK activation may be detrimental in HD, with increased levels being associated with oxidative stress and brain atrophy (82, 83). Specifically, it was found that AICAR administration into the striatum *via* subcutaneous osmotic pumps exacerbated the presence of reactive oxygen species in R6/2 mice (82). Additionally, these intra-striatal AICAR injections were associated with increased brain atrophy, neuronal loss, and mHTT aggregate formation (83). In contrast, oral treatment with metformin, a widely used antidiabetic agent known to activate AMPK, improved survival, and certain motor deficits in R6/2 mice (44). In a murine model of early HD, striatal injection of a lentivirus overexpressing AMPK decreased lesion size. A similar construct overexpressed in primary striatal cell cultures from HD mice reduced cell mortality and levels of soluble mHTT (42). Based on these studies, AMPK modulation at the level of the CNS appears to be an interesting avenue for pharmacological interventions for HD.

Our experiments were specifically designed for peripheral delivery of AICAR. As AICAR only crosses the blood–brain barrier in negligible amounts (43, 46), we hypothesized that altering the muscle phenotype through EM treatment would confer benefits to the brain through peripheral mechanisms. However, under our treatment conditions, motor coordination and locomotion were unaffected by AICAR treatment as tested by rotarod and open field tests, respectively. Specifically, HD mice had a shorter latency to fall from the accelerating rotarod and traveled a shorter distance in the open field compared with their WT counterparts. This phenotypic presentation worsened with age, and is corroborated in the existing literature (50, 52).

Previous studies have shown beneficial neurological effects of peripheral AICAR injections in mice. Indeed, AICAR improved performance in the Morris water maze, an effect that was nullified in muscle-specific AMPK-inactivated mice (47, 48). Peripheral AICAR injections also upregulated hippocampal neurogenesis, particularly short-term treatments (38, 48). Therefore, the lack of positive effects of AICAR on HD progression seen in our study indicates that our AICAR treatment conditions were insufficient to mitigate HD disease progression in R6/2 mice.

Huntington’s disease is known to affect multiple key cellular processes, as mHTT is expressed in many tissues (84). mHTT has been shown to form aggregates in skeletal muscle of mouse models of HD (85, 86) as well as in primary muscle cell cultures from HD patients (87). The present work shows the presence of mHTT-positive puncta in R6/2 mouse skeletal muscle. In addition, mHTT aggregates were observed the striatum of our mice. As with the behavioral presentation of HD in our mouse model, these disease markers were unaffected by AICAR treatment. Striatal mHTT aggregates have been described as both protective and detrimental (88), and little work has been done on the accumulation of mHTT in muscle (84). As such, more work is required to understand the importance of mHTT aggregation and mechanisms promoting their clearance in both skeletal muscle and neurons.

## Conclusion

Under our experimental conditions, our data indicate that chronic administration of AICAR failed to affect HD progression in R6/2 mice. However, AICAR successfully induced a muscle phenotypic switch in HD mice leading to a greater oxidative metabolism, which, by itself, may prove beneficial for patient populations with limited mobility. Accordingly, additional studies to examine the potential benefits of different treatment paradigms and other EMs in HD seem warranted.

## Ethics Statement

All animal procedures were approved by the University of Ottawa Animal Care Committee and were in accordance with the Canadian Council of Animal Care Guidelines.

## Author Contributions

MFP and BJ conceived and designed the experiments, and wrote the manuscript. MFP performed all experiments and analyzed the data. BJ contributed reagents, materials, and analysis tools.

## Conflict of Interest Statement

The authors declare that the research was conducted in the absence of any commercial or financial relationships that could be construed as a potential conflict of interest.
